# Role of Particle Entanglement in the Violation of Bell Inequalities

**DOI:** 10.1038/s41598-018-20034-8

**Published:** 2018-01-29

**Authors:** Tomasz Wasak, Augusto Smerzi, Jan Chwedeńczuk

**Affiliations:** 1 Faculty of Physics, University of Warsaw, ul. Pasteura 5, PL–02–093 Warszawa, Poland; 2 QSTAR, INO-CNR and LENS, Largo Enrico Fermi 2, 50125 Firenze, Italy

## Abstract

Entanglement between two separate systems is a necessary resource to violate a Bell inequality in a test of local realism. We demonstrate that to overcome the Bell bound, this correlation must be accompanied by the entanglement between the constituent particles. This happens whenever a super-selection rule prohibits coherences between states with different total number of particles and thus imposes a constraint on feasible local operations in each sub-system. We show that the necessary entanglement between the particles might solely result from their indistinguishability. We also give an example of both mode and particle-entangled pure state, which does not violate any Bell inequality. Our result reveals a fundamental relation between the non-locality and the particle entanglement.

## Introduction

The “spooky action at the distance” stands out among the most striking consequences of quantum mechanics^1^. This term was coined by Albert Einstein to underline how counterintuitive it is that a seemingly local manipulation on one part of a system immediately affects its other distant part without any signal transferred from one subsystem to the other. Such an effect contradicts the postulates of the “local realism”: a pair of spatially separated, entangled particles can show correlations that cannot be reproduced by any local, hidden variable theory. The non-locality of quantum mechanics can be revealed by a series of inequalities—first considered by Bell^2^—for the correlations between the outcomes of local measurements^3–19^. The violation of the Bell inequalities was first observed long ago^5–8^ and since then large efforts, devoted to improve the experimental techniques^20^, culminated in the recent “loophole-free” deviation from local realism claimed in^19,21,22^.

Not all entangled states violate a (known) Bell inequality^23^, but it has been shown that all entangled *pure* states do violate a Bell inequality^24^. For illustration, consider a pure state |***ψ***〉 shared by two parties *A* and *B* that are spatially separated (so that all physical manipulations and measurements performed by one party cannot affect the other one by classical communication or local operations):

1 $$|{\boldsymbol{\psi }}\rangle =\sum _{i}{c}_{i}|{{\varphi }}_{i}{\rangle }_{A}\otimes |{{\chi }}_{i}{\rangle }_{B},$$

where the state has been written in Schmidt decomposition on orthonormal bases of the parties *A* and *B*, respectively. If the state is *A*-*B* entangled—which happens when at least two coefficients of this expansion, say *c*_*i*_ and *c*_*i*′_, are non-zero—a Bell inequality^24^ will be violated by locally coupling $|{{\varphi }}_{i}{\rangle }_{A}$ with $|{{\varphi }}_{i}^{\prime} {\rangle }_{A}$| and |*χ*_*i*_〉_*B*_ with |*χ*_*i*′_〉_*B*_.

However, sometimes local operations and/or measurements are prohibited by some superselection rule (SSR). The SSR is a restriction imposed on quantum mechanics forbidding coherences between eigenstates of certain observables^25,26^. For the purpose of this manuscript, the SSR can be formulated as follows: local operations/measurements cannot create/detect coherences between states with different number of particles. Here, a particle is understood as a discrete object carrying a set of fundamental quantum numbers, such as the charge or the baryon and lepton numbers^27^.

To illustrate the impact of the SSR on the feasible local operations, consider two states localized in *A*: one which contains a single sodium 23 atom, denoted by |^23^Na〉_*A*_ and the other with a rubidium 87 atom, denoted by |^87^Rb〉_*A*_. Although these states have the same number of atoms, any operation or measurement coupling these states would not preserve the number of baryons. Therefore, such coupling is forbidden by the SSR^25,26,28^ imposing the conservation of the total number of baryons. Another known example is a single particle in the superimposed state^29^:

2 $$|{\boldsymbol{\psi }}\rangle =\frac{1}{\sqrt{2}}(\mathrm{|1}{\rangle }_{A}\otimes \mathrm{|0}{\rangle }_{B}+\mathrm{|0}{\rangle }_{A}\otimes \mathrm{|1}{\rangle }_{B}).$$

The SSR formulated above prohibits the local creation or detection of a superposition of the vacuum |0〉_*A*_ with the state containing one particle |1〉_*A*_. From the point of view of physically realizable local operations one can effectively replace the pure state (2) with an incoherent mixture

3 $$|{\boldsymbol{\psi }}\rangle \langle {\boldsymbol{\psi }}|\to {\hat{\rho }}_{{\rm{e}}ff}=\frac{1}{2}(\mathrm{|1}\rangle \langle {\mathrm{1|}}_{A}\otimes \mathrm{|0}\rangle \langle {\mathrm{0|}}_{B}+\mathrm{|0}\rangle \langle {\mathrm{0|}}_{A}\otimes \mathrm{|1}\rangle \langle {\mathrm{1|}}_{B}).$$

Although the state (2) is *A*-*B* entangled, due to the SSR the resulting ${\hat{\rho }}_{{\rm{e}}ff}$ is *A*-*B* separable (i.e., non-entangled) and as such does not violate any Bell inequality^27,30–32^. Note that for photons, to which the SSR does not apply, the local coupling of |0〉_*A*_ with |1〉_*A*_ is allowed and indeed the state (2) violates a Bell inequality^19,21,29^.

Inspired by this example we formulate and prove a general theorem: the restriction imposed on the local operations by the SSR renders not only the *A*-*B* entanglement but also the entanglement of particles shared by *A* and *B* necessary for the violation of any Bell inequality. We demonstrate that this latter resource might origin solely from the particle indistinguishability^33^.

## Results

To set the stage and proceed with the proof, we note that the *A*-*B* separable states have a general form

4 $$\hat{\rho }=\sum _{i}\,{p}_{i}\,{\hat{\rho }}_{A}^{(i)}\otimes {\hat{\rho }}_{B}^{(i)},$$

where *p*_*i*_’s are the statistical weights. Here, ${\hat{\rho }}_{A}^{(i)}$ and ${\hat{\rho }}_{B}^{(i)}$ represent the quantum state of the subsystems in *A* and *B*, respectively. We will demonstrate that in presence of SSR and in the context of Bell inequalities, the quantum state should also be inspected through the relation between the *N* particles shared by *A* and *B*. Particle-separable states can be written as

5 $$\hat{\rho }=\sum _{i}\,{\tilde{p}}_{i}\,{\hat{\rho }}_{i}^{\mathrm{(1)}}\otimes \ldots \otimes {\hat{\rho }}_{i}^{(N)},$$

where ${\hat{\rho }}_{i}^{(n)}$ is a quantum state of the *n*-th particle. The particle-entangled states are those that cannot be written in this way. We show that all particle separable states do not violate any Bell inequality in presence of SSR. In other words, the quantum state shared by *A* and *B* must necessarily be particle-entangled to violate any Bell inequality. Note that the SSR imposes restrictions both globally (on the whole state) and locally, in *A* and in *B* separately: it prohibits coherences between states with different particle numbers and renders some local operations unphysical.

### Distinguishable particles

First, we consider a collection of distinguishable particles. The basic building block of the *N*-body density matrix (5) is the one-body pure state, which for the *i*-th particle reads

6 $$|{{\boldsymbol{\psi }}}_{i}\rangle =(\alpha ({{\boldsymbol{\psi }}}_{i}){\hat{{\boldsymbol{\psi }}}}_{i}^{(A)\dagger }+\beta ({{\boldsymbol{\psi }}}_{i})\,{\hat{{\boldsymbol{\psi }}}}_{i}^{(B)\dagger })\mathrm{|0}\rangle .$$

Here ${\hat{{\boldsymbol{\psi }}}}_{i}^{(k)\dagger }$ creates a quantum of a field associated with this particle in the region *k*, |0〉 is the vacuum and |*α*(***ψ***_*i*_)|^2^ + |*β*(***ψ***_*i*_)|^2^ = 1. According to Eq. (5), the density matrix of *N* particles forming a separable state is an incoherent mixture of the one-body matrices:

7 $$\hat{\rho }=\int {\mathscr{D}}{{\boldsymbol{\psi }}}_{1}\cdots \int {\mathscr{D}}{{\boldsymbol{\psi }}}_{N}\,{\mathscr{P}}({{\boldsymbol{\psi }}}_{1},\ldots ,{{\boldsymbol{\psi }}}_{N})\underset{i=1}{\overset{N}{\otimes }}|{{\boldsymbol{\psi }}}_{i}\rangle \langle {{\boldsymbol{\psi }}}_{i}\mathrm{|}.$$

Here, the joint probability ${\mathscr{P}}({{\boldsymbol{\psi }}}_{1},\ldots ,{{\boldsymbol{\psi }}}_{N})$ determines the partition of all the particles among *A* and *B*. The symbol ${\mathscr{D}}{\psi }_{i}$ is the integration measure over the set of fields ${{\boldsymbol{\psi }}}_{i}$.

Following the example from Eq. (2), the SSR enforces every |***ψ***_*i*_〉〈***ψ***_*i*_| to be replaced with

8 $$|{{\boldsymbol{\psi }}}_{i}\rangle \langle {{\boldsymbol{\psi }}}_{i}|\to {\hat{\rho }}_{{\rm{e}}ff}({{\boldsymbol{\psi }}}_{i})=|\alpha ({{\boldsymbol{\psi }}}_{i}{)|}^{2}{\hat{{\boldsymbol{\psi }}}}_{i}^{(A)\dagger }\mathrm{|0}\rangle \langle \mathrm{0|}\,{\hat{{\boldsymbol{\psi }}}}_{i}^{(A)}+|\beta ({{\boldsymbol{\psi }}}_{i}{)|}^{2}{\hat{{\boldsymbol{\psi }}}}_{i}^{(B)\dagger }\mathrm{|0}\rangle \langle \mathrm{0|}\,{\hat{{\boldsymbol{\psi }}}}_{i}^{(B)}.$$

This expression, plugged back into (7) gives

9 $${\hat{\rho }}_{{\rm{e}}ff}=\int {\mathscr{D}}{{\boldsymbol{\psi }}}_{1}\cdots \int {\mathscr{D}}{{\boldsymbol{\psi }}}_{N}\,{\mathscr{P}}({{\boldsymbol{\psi }}}_{1},\ldots ,{{\boldsymbol{\psi }}}_{N})\underset{i=1}{\overset{N}{\otimes }}{\hat{\rho }}_{{\rm{e}}ff}({{\boldsymbol{\psi }}}_{i}\mathrm{)}.$$

Since the inter-region coherence is washed out already on the particle level of Eq. (8) and the integral over the fields does not introduce any quantum coherence, the effective *N*-body density matrix ${\hat{\rho }}_{{\rm{e}}ff}$ is both particle- and *A*-*B*-separable (for the rigorous proof, see Methods). To conclude, the SSR “transforms” the state (7) into (9), which has the form of Eq. (4), and as such it will not violate any Bell inequality.

If the distinguishable particles are entangled, violation of Bell inequality in presence of SSR might be possible. For illustration, consider an electron (*e*) and a proton (*p*) forming a particle- and *A*-*B*-entangled state

10 $$|{\boldsymbol{\psi }}\rangle =\frac{1}{\sqrt{2}}({|{\uparrow }_{{e}}\rangle }_{{A}}\otimes {|{\uparrow }_{{p}}\rangle }_{B}+{|{\downarrow }_{e}\rangle }_{A}\otimes {|{\downarrow }_{{p}}\rangle }_{B}),$$

where the arrows denote the projection of the spin of each particle on some quantization axis. Now, local operations can be executed by coupling |↑_*e*_〉_*A*_ with |↓_*e*_〉_*A*_ and |↑_*p*_〉_*B*_ with |↓_*p*_〉_*B*_. Therefore, according to the discussion below Eq. (1), this state will violate a Bell inequality. On the other hand, take an alternative particle- and *A*-*B*-entangled state

11 $$|{\boldsymbol{\psi }}\rangle =\frac{1}{\sqrt{2}}({|{\uparrow }_{e},{\uparrow }_{p}\rangle }_{A}\otimes {|0\rangle }_{B}+{|0\rangle }_{A}\otimes {|{\downarrow }_{e},{\downarrow }_{p}\rangle }_{B}).$$

It will not violate any Bell inequality, because SSR forbid the coupling of |↑_*e*_, ↑_*p*_〉_*A*_ with |0〉_*A*_ and |↓_*e*_, ↓_*p*_〉_*B*_ with |0〉_*B*_. This second example highlights the fact that when the SSR apply, both the particle and the *A*-*B* entanglement are only necessary, but not sufficient to drive the violation of a Bell inequality.

### Indistinguishable particles

We now turn to bosons (for a comprehensive review of the entanglement criteria for bosons, see^34–36^), for which a separable state can be written, in analogy with Eq. (7), as^37,38^

12 $$\hat{\rho }=\int {\mathscr{D}}{\boldsymbol{\psi }}\,{\mathscr{P}}({\boldsymbol{\psi }})|{\boldsymbol{\psi }}\rangle \langle {\boldsymbol{\psi }}\mathrm{|}.$$

Here $|{\boldsymbol{\psi }}\rangle$ is the spin coherent state, which reads

13 $$|{\boldsymbol{\psi }}\rangle =\frac{1}{\sqrt{N!}}{(\alpha ({\boldsymbol{\psi }}){\hat{{\boldsymbol{\psi }}}}^{(A)\dagger }+\beta ({\boldsymbol{\psi }}){\hat{{\boldsymbol{\psi }}}}^{(B)\dagger })}^{N}|0\rangle .$$

The language of the second quantization allows to immediately identify the relation between *A* and *B*, i.e, provides the state decomposed as in Eq. (4). This can be seen by writing Eq. (13) in terms of *A*/*B* occupation states, i.e,

14 $$|{\boldsymbol{\psi }}\rangle =\sum _{{n}_{{\boldsymbol{\psi }}}=0}^{N}{C}_{{n}_{{\boldsymbol{\psi }}}}|{n}_{{\boldsymbol{\psi }}}{\rangle }_{A}\otimes |N-{n}_{{\boldsymbol{\psi }}}{\rangle }_{B},$$

where $${C}_{n}=\sqrt{(\begin{array}{c}N\\ n\end{array})}\alpha {({\boldsymbol{\psi }})}^{n}\beta {({\boldsymbol{\psi }})}^{N-n}$$. This expression plugged into Eq. (12) gives

15 $$\hat{\rho }=\int {\mathscr{D}}{\boldsymbol{\psi }}\,{\mathscr{P}}({\boldsymbol{\psi }})\sum _{{n}_{{\boldsymbol{\psi }}}=0}^{N}\sum _{{m}_{{\boldsymbol{\psi }}}=0}^{N}{C}_{{n}_{{\boldsymbol{\psi }}}}^{\ast }{C}_{{m}_{{\boldsymbol{\psi }}}}|{n}_{{\boldsymbol{\psi }}}\rangle \langle {m}_{{\boldsymbol{\psi }}}{|}_{A}\otimes |N-{n}_{{\boldsymbol{\psi }}}\rangle \langle N-{m}_{{\boldsymbol{\psi }}}{|}_{B}.$$

In presence of SSR, local operations cannot couple |*n*〉_*k*_ with |*n*′〉_*k*_. In this context, the state ((14)) can be effectively replaced by

16 $$|{\boldsymbol{\psi }}\rangle \langle {\boldsymbol{\psi }}|\to {\hat{\rho }}_{{\rm{e}}ff}({\boldsymbol{\psi }})=\sum _{{n}_{{\boldsymbol{\psi }}}=0}^{N}|{C}_{{n}_{{\boldsymbol{\psi }}}}{|}^{2}|{n}_{{\boldsymbol{\psi }}}\rangle \langle {n}_{{\boldsymbol{\psi }}}{|}_{A}\otimes |N-{n}_{{\boldsymbol{\psi }}}\rangle \langle N-{n}_{{\boldsymbol{\psi }}}{|}_{B},$$

which is both particle- and *A*-*B*-separable. Also the effective density matrix

17 $${\hat{\rho }}_{{\rm{e}}ff}=\int {\mathscr{D}}{\boldsymbol{\psi }}{\mathscr{P}}({\boldsymbol{\psi }}){\hat{\rho }}_{{\rm{e}}ff}({\boldsymbol{\psi }})$$

is *A*-*B* separable. Thus, for the same argument illustrated in the previous Section, in presence of SSR the particle entanglement is a necessary resource for the violation of any Bell inequality.

### Example

We now show that the entanglement extracted solely from the indistinguishability of bosons might be sufficient for the violation of the Bell inequality^39–42^.

**Figure 1:**
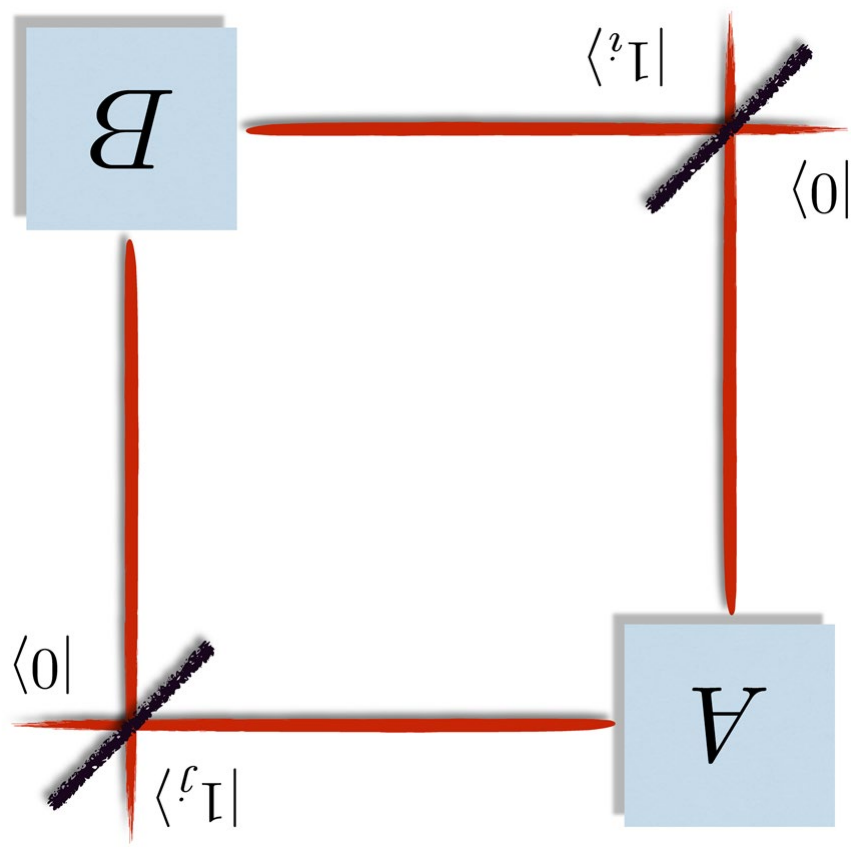
Two particles are coherently split and sent into distant parties *A* and *B*. If the particles are distinguishable, the system will not violate any Bell inequality in presence of SSR, because the state shared by *A* and *B* is effectively particle-separable. If the particles are identical, entanglement due to (anti-) symmetrization is sufficient to violate a Bell ineqality.

Consider one a particle of type *i* and a particle of type *j* in a state

18 $$|{\boldsymbol{\psi }}\rangle ={\mathrm{|1}}_{i}\rangle \otimes {\mathrm{|1}}_{j}\rangle $$

entering the system through the two ports^43^, shown in Fig. 1. The two beam-splitters distribute the signal among *A* and *B*,

19 $$|{\boldsymbol{\psi }}^{\prime} \rangle =\frac{1}{2}{{\mathrm{(|1}}_{i}\rangle }_{A}+{\mathrm{|1}}_{i}{\rangle }_{B})\otimes {{\mathrm{(|1}}_{j}\rangle }_{A}+{\mathrm{|1}}_{j}{\rangle }_{B}\mathrm{)}.$$

The symbol ⊗ in Equations (18) and (19) multiplies the single-particle states, but to analyze the relation between *A* and *B*, one should switch to the second-quantization. This is obtained by expanding the product and expressing |***ψ***〉 in terms of *A*- and *B*-occupation states. For instance, |1_*i*_〉_*A*_⊗|1_*j*_〉_*B*_ → |1_*i*_,0_*j*_〉_*A*_⊗|0_*i*_,1_*j*_〉_*B*_, giving

20 $$|{\boldsymbol{\psi }}^{\prime} \rangle =\frac{1}{2}{{\mathrm{(|1}}_{i}{\mathrm{,0}}_{j}\rangle }_{A}\otimes {\mathrm{|0}}_{i}{\mathrm{,1}}_{j}{\rangle }_{B}+{\mathrm{|0}}_{i}{\mathrm{,1}}_{j}{\rangle }_{A}\otimes {\mathrm{|1}}_{i}{\mathrm{,0}}_{j}{\rangle }_{B}+{\mathrm{|1}}_{i}{\mathrm{,1}}_{j}{\rangle }_{A}\otimes \mathrm{|0,0}{\rangle }_{B}+\mathrm{|0,0}{\rangle }_{A}\otimes {\mathrm{|1}}_{i}{\mathrm{,1}}_{j}{\rangle }_{B}\mathrm{)}.$$

Now it is clear that the state is *A*-*B* entangled. If the particles are distinguishable, i.e., *i* ≠ *j*, they are not entangled and the only pair of states in *A* with equal number of particles are |1_*i*_,0_*j*_〉_*A*_ and |0_*i*_,1_*j*_〉_*A*_ (and analogically in *B*). These states cannot be locally coupled in presence of SSR and the system will not violate any Bell inequality. On the other hand, if the particles are identical, *i* and *j* labels the two ports (modes) through which a pair of indistinguishable particles enters the system. In this case, the state (18) is particle-entangled state due to the indistinguishability and after the splitting it reads

21 $$|{\boldsymbol{\psi }}^{\prime} \rangle =\frac{1}{2}(\mathrm{|1,0}{\rangle }_{A}\otimes \mathrm{|0,1}{\rangle }_{B}+\mathrm{|0,1}{\rangle }_{A}\otimes \mathrm{|1,0}{\rangle }_{B}+\mathrm{|1,1}{\rangle }_{A}\otimes \mathrm{|0,0}{\rangle }_{B}+\mathrm{|0,0}{\rangle }_{A}\otimes \mathrm{|1,1}{\rangle }_{B}).$$

Now, the coupling of |1,0〉_*k*_ with |0,1〉_*k*_ can be realized, and particle entanglement coming solely from indistinguishability^33^ will drive the violation of some Bell inequality.

## Discussion

If a separable state contains a group of bosons and a group of distinguishable particles, all above arguments can be applied to each subgroup separately, since local operations, in presence of SSR prohibit the transmutation of a particle of one type into another. Moreover, if the state reveals incoherent particle-number fluctuations, that are consistent with SSR, each fixed-*N* sector can be considered separately, leading to the same conclusion—particle-separable states do not reveal non-locality in any Bell test. One could extend the system by adding an auxiliary reference frame to the particle-separable state^44,45^. If this reference frame is quantum and obeys the SSR, then, according to our proof, as long as this extension does not introduce any particle entanglement, the composite system will remain effectively *A*-*B* separable.

Finally, we point that the mode- and the particle-entanglement, together forming a set of necessary conditions to observe the violation of the Bell inequality, are also a resource in quantum interferometry. There, the sub-shot noise sensitivity of the phase estimation can be achieved only if these two types of correlations are present during the imprint of the interferometric phase^46,47^.

To summarize, we have shown that in presence of super-selection rules, mode entanglement must be accompanied by entanglement between the particles in order to violate a Bell inequality. This is the case for distinguishable particles, bosons, or systems where bosons and distinguishable particles co-exist. Our result puts the particle entanglement on par with the *A*-*B* mode entanglement, as a necessary condition for the violation of the local realism. We have demonstrated that the particle entanglement necessary for the violation of the Bell inequalities might result solely from the indistinguishability of bosons. An example of a pure state which is both *A*-*B* and particle-entangled, but due to the SSR does not violate any Bell inequality, underlines that the presence of both these types of correlations is merely a necessary condition.

## Methods

Here we present the rigorous proof of the transformation from Eq. (7) to the Eq. (9) in presence of SSR. To this end, note that the one-body pure state for the particle of type *i*, which is distributed among the the parties *A* and *B* reads

22 $$|{{\boldsymbol{\psi }}}_{i}\rangle =(\alpha ({{\boldsymbol{\psi }}}_{i}){\hat{{\boldsymbol{\psi }}}}_{i}^{(A)\dagger }+\beta ({{\boldsymbol{\psi }}}_{i}){\hat{{\boldsymbol{\psi }}}}_{i}^{(B)\dagger })|0\rangle .$$

We introduce a shortened notation, where

23 $$\alpha ({{\boldsymbol{\psi }}}_{i}){\hat{{\boldsymbol{\psi }}}}_{i}^{(A)\dagger }\equiv {\hat{{\rm{\Phi }}}}_{i}^{(A)\dagger }\quad {\rm{a}}{\rm{n}}{\rm{d}}\quad \beta ({{\boldsymbol{\psi }}}_{i}){\hat{{\boldsymbol{\psi }}}}_{i}^{(B)\dagger }\equiv {\hat{{\rm{\Phi }}}}_{i}^{(B)\dagger }.$$

With this at hand, the state (22) is

24 $$|{{\boldsymbol{\psi }}}_{i}\rangle =\sum _{{\kappa }_{i}\in \{A,B\}}{\hat{{\rm{\Phi }}}}_{i}^{({\kappa }_{i})\dagger }|0\rangle .$$

Every density matrix of *N* particles in a separable state can be expressed as

25 $$\hat{\rho }=\int {\mathscr{D}}{{\boldsymbol{\psi }}}_{1}\cdots \int {\mathscr{D}}{{\boldsymbol{\psi }}}_{N}\,{\mathscr{P}}({{\boldsymbol{\psi }}}_{1},\ldots ,{{\boldsymbol{\psi }}}_{N})\underset{i=1}{\overset{N}{\otimes }}|{{\boldsymbol{\psi }}}_{i}\rangle \langle {{\boldsymbol{\psi }}}_{i}|,$$

where ${\mathscr{P}}({{\boldsymbol{\psi }}}_{1},\ldots ,{{\boldsymbol{\psi }}}_{N})$ is a probability distribution. We now insert the expression (24) into (25) and obtain

26 $$\hat{\rho }=\sum _{{\kappa }_{1}\in \{A,B\}}\cdots \sum _{{\kappa }_{N}\in \{A,B\}}\sum _{{\kappa ^{\prime} }_{1}\in \{A,B\}}\cdots \sum _{{\kappa ^{\prime} }_{\mathrm{N}}\in \{A,B\}}\int {\mathscr{D}}{{\boldsymbol{\psi }}}_{1}\cdots \int {\mathscr{D}}{{\boldsymbol{\psi }}}_{N}\,{\mathscr{P}}({{\boldsymbol{\psi }}}_{1},\ldots ,{{\boldsymbol{\psi }}}_{N}){\hat{{\rm{\Phi }}}}_{1}^{({\kappa }_{1})\dagger }\cdots {\hat{{\rm{\Phi }}}}_{N}^{({\kappa }_{N})\dagger }|0\rangle \langle 0|{\hat{{\rm{\Phi }}}}_{1}^{({\kappa ^{\prime} }_{1})}\cdots {\hat{{\rm{\Phi }}}}_{N}^{({\kappa ^{\prime} }_{\mathrm{N}})}.$$

In this state, the quantum correlation between the particles in *A* and *B* arises from the one-body coherence, which is represented in the independent sums over *κ*_*i*_ and *κ*′_*i*_. The restriction imposed on local operations require that *A* and *B* can only couple states with a fixed number of particles obeying the SSR. This means that the sums over *κ*_*i*_ and *κ*′_*i*_ effectively do not run independently, and the state reduces to

27 $${\hat{\rho }}_{{\rm{e}}ff}=\sum _{{\kappa }_{1}\in \{A,B\}}\cdots \sum _{{\kappa }_{N}\in \{A,B\}}\int {\mathscr{D}}{{\boldsymbol{\psi }}}_{1}\cdots \int {\mathscr{D}}{{\boldsymbol{\psi }}}_{N}\,{\mathscr{P}}({{\boldsymbol{\psi }}}_{1},\ldots ,{{\boldsymbol{\psi }}}_{N}){\hat{{\rm{\Phi }}}}_{1}^{({\kappa }_{1})\dagger }\cdots {\hat{{\rm{\Phi }}}}_{N}^{({\kappa }_{N})\dagger }|0\rangle \langle 0|{\hat{{\rm{\Phi }}}}_{1}^{({\kappa }_{1})}\cdots {\hat{{\rm{\Phi }}}}_{N}^{({\kappa }_{N})}.$$

This state does not reveal any quantum coherence and is *A*-*B* separable.               $\square$
